# Estimating Need for Glasses and Hearing Aids in The Gambia: Results from a National Survey and Comparison of Clinical Impairment and Self-Report Assessment Approaches

**DOI:** 10.3390/ijerph18126302

**Published:** 2021-06-10

**Authors:** Dorothy Boggs, Abba Hydara, Yaka Faal, John Atta Okoh, Segun Isaac Olaniyan, Haruna Sanneh, Abdoulie Ngett, Isatou Bah, Mildred Aleser, Erima Denis, Ian McCormick, Tess Bright, Suzannah Bell, Minjung Kim, Allen Foster, Hannah Kuper, Matthew J. Burton, Islay Mactaggart, Sarah Polack

**Affiliations:** 1 International Centre for Evidence in Disability, London School of Hygiene & Tropical Medicine, London WC1E 7HT, UK; Tess.Bright@lshtm.ac.uk (T.B.); Hannah.kuper@lshtm.ac.uk (H.K.); islay.mactaggart@lshtm.ac.uk (I.M.); Sarah.Polack@lshtm.ac.uk (S.P.); 2 Sheikh Zayed Regional Eye Care Centre, Kanifing, The Gambia; ahydara@gmail.com (A.H.); okohaj@yahoo.com (J.A.O.); segstar007@gmail.com (S.I.O.); harunatssanneh@yahoo.com (H.S.); ngett10@yahoo.com (A.N.); ndeysatou@yahoo.com (I.B.); msgbireh@yahoo.com (M.A.); 3 Ear Nose and Throat Unit, Edward Francis Small Teaching Hospital, Banjul, The Gambia; yaka.faal@yahoo.com; 4 Lubaga Hospital, Kampala, Uganda; deni-remas@hotmail.com; 5 International Centre for Eye Health, London School of Hygiene & Tropical Medicine, London WC1E 7HT, UK; Ian.McCormick@lshtm.ac.uk (I.M.); Suzannah.bell@nhs.net (S.B.); Min.Kim@lshtm.ac.uk (M.K.); Allen.Foster@lshtm.ac.uk (A.F.); Matthew.Burton@lshtm.ac.uk (M.J.B.); 6 National Institute for Health Research Biomedical Research Centre for Ophthalmology at Moorfields Eye Hospital NHS Foundation Trust and UCL Institute of Ophthalmology, London EC1V 9EL, UK

**Keywords:** assistive device, surveys, need, access, glasses, hearing aids, vision impairment, hearing impairment

## Abstract

Few estimates are available of the need for assistive devices (ADs) in African settings. This study aimed to estimate population-level need for glasses and hearing aids in The Gambia based on (1) clinical impairment assessment, and (2) self-reported AD awareness, and explore the relationship between the two methods. The Gambia 2019 National Eye Health Survey is a nationally representative population-based sample of 9188 adults aged 35+ years. Participants underwent standardised clinical vision assessments including the need for glasses (distance and near). Approximately 25% of the sample underwent clinical assessment of hearing and hearing aid need. Data were also collected on self-reported awareness, need and access barriers to vision and hearing ADs. Overall, 5.6% of the study population needed distance glasses (95% CI 5.0–6.3), 45.9% (95% CI 44.2–47.5) needed near glasses and 25.5% (95% CI 22.2–29.2) needed hearing aids. Coverage for each AD was very low (<4%). The agreement between self-report and clinical impairment assessment for AD need was poor. In conclusion, there is high prevalence and very low coverage for distance glasses, near glasses and hearing aids in The Gambia. Self-report measures alone will not provide an accurate estimate of AD need.

## 1. Introduction

Globally, the World Health Organization (WHO) estimates there are at least one billion people in need of assistive technology (AT). AT includes both assistive devices (ADs) and the systems and services related to AD delivery [1,2]. AT users can include older people, people with disabilities, and people living with chronic health conditions, non-communicable diseases, and communicable diseases [1]. Global AT need is expected to rise to 2 billion by 2030, given population ageing and the increase in non-communicable disease prevalence; however, reliable data on AT need are scarce [2]. For example, the global need estimate is based upon extrapolations of global burden of disease (GBD) data, and data are especially limited regarding need for individual ADs.

There has been an increase in global initiatives to scale up AT access over the past ten years, including the WHO Global Cooperation on Assistive Technology (GATE) initiative [3] and ATScale [4]. GATE developed a global priority assistive products list (APL) which includes over 50 ADs, and five of these were selected as ATScale priority ADs: glasses, hearing aids, wheelchairs, prosthetics, and digital AT [1,4]. However, reliable data on population-level AD need, coverage and access are required for evidence-based advocacy and planning of programmes to increase provision and access to AT. To address the AT data gap, WHO GATE developed the rapid Assessment of Assistive Technology (rATA), a population-based survey measuring self-reported use, need and barriers to accessing AT for over 30 priority ADs across the six functional domains of vision, hearing, mobility, communication, cognition and self-care [5]. The rATA is relatively rapid, low-cost to administer and collects data in a standardised way; however, the rATA uses self-reported AD measurement only.

There are other approaches to measuring AT need which differ in the way impairment, AT and functioning are defined, conceptualised and measured. For example, clinical impairment assessment (e.g., visual acuity measurement) uses standardised clinical methods to assess the presence of impairment followed by a clinician’s assessment of AD needs (e.g., glasses) based on type, cause and severity of the impairment. Clinical assessment provides important information on whether AD or medical treatment is needed, but may be costly to administer, often requires input from clinical staff and may not take into account the participant’s perceptions/environment. Previous analysis of surveys in India and Cameroon suggested substantial discrepancy between self-report and clinical assessment approaches [6,7]. However, the sample sizes for these analyses were relatively small and gaps were identified, including a lack of consistency in the collection of data on AD need (for example, standardised AD definitions and pictorial aids were lacking) and disaggregation of AD need by impairment severity [6]. In this paper, we address these gaps and provide further comparisons between self-report and clinical impairment assessment approaches for assessing need for glasses (distance and near) and hearing aids in The Gambia.

The Gambia is a small country in western Africa with a population of 2,335,000 and life expectancy of 62 years in 2018 [8,9]. Given the increase in the proportion of the population who are older, alongside a rise in migration from rural to urban areas and in non-communicable diseases, it is likely that the population will have increasing AT needs; however, data are lacking about impairment prevalence and related functional service needs in this population [10]. In 2019, a National Eye Health Survey was conducted in The Gambia to estimate the prevalence and causes of vision impairment, blindness and its comorbidities [11]. This involved clinical vision assessment of a nationally representative population-based sample of adults 35 years and above, as well as data collection on comorbidities, including hearing impairment, disability, and need for and access to related vision and hearing ADs [11].

In this paper, we conduct an analysis of national-level survey data in The Gambia in adults 35 years and above in order to:

1. Estimate population-level total need, unmet need and coverage for glasses (distance and near glasses) and hearing aids, two of the five ATScale priority ADs.
2. Estimate reported AD awareness, need and access barriers.
3. Explore the relationship between clinical impairment and self-report assessment methods for assessing AD need within population surveys.

## 2. Methods

A National Eye Health survey was conducted in The Gambia in adults 35 years and over from February to July 2019. Hearing assessments were completed in clusters visited by one of the four teams which equated to approximately one-quarter of participants.

The methodology of the full survey is published elsewhere [11]. Using the 2013 census data as the sample frame, multi-stage stratified cluster random sampling with probability proportional to size (PPS) procedures were used to identify a nationally representative sample of adults 35 years and older. The survey was powered to detect eye disease prevalence as low as 0.5%. This required an overall sample size of 10,800 adults 35 years and older in 360 clusters of approximately 30 adults per cluster, assuming an intraclass correlation coefficient of 0.038, a design effect of 2.5, a 20% non-response rate and a margin of error of 20% around the estimate. For the hearing component, the target sample size was 2700 (1/4 overall sample) which was powered to detect a 9% prevalence of hearing impairment [12]. Hearing assessment was conducted in adults aged 35+ in approximately one-quarter of the clusters (90/360 clusters) by one of the four survey teams.

Standard Gambia Bureau of Statistics (GBoS) Census Enumeration Areas (EAs) were used as clusters, and the 360 clusters were randomly selected via PPS. For each cluster, enumerators first undertook a household listing of eligible participants. Using this information, the cluster was then subdivided into segments each including approximately 30 adults aged 35+. One segment was randomly selected, and all adults in the selected segment were invited to a central location the following day for clinical assessments.

Data were collected in a central location on mobile tablets using Open Data Kit (ODK). There were four survey teams each comprised of one ophthalmologist, one optometrist or optometry technician, one senior ophthalmic medical assistant (SOMA), one general nurse, one mental health nurse, and two enumerators. In one team, the one practicing audiology nurse in The Gambia was included. Teams underwent ten days of training which included standardised tests of protocol adherence, practice examinations and pilot testing. Questionnaires were pre-tested and revised where necessary following the pilot. A formal interobserver variability test was completed for vision testing only, with kappa agreement of 0.7 and 0.8 for two teams, while one team achieved a fairly low agreement (0.4) requiring further training review before data collection [11].

All participants completed a general demographic and socioeconomic questionnaire which included use of the EquityTool, an objective tool comprised of 12 country-specific assets that was used to generate a wealth index [13]. All study materials, including the questionnaires, are presented in Supplementary File S1.

### 2.1. Self-Reported Functioning and AD Awareness, Need and Access Barriers

Before the clinical assessments, data on self-reported level of difficulty in seeing and hearing were collected on all participants using the relevant questions from a modified version of the Washington Group Short Set questions [14,15]. These questions use a four-point response scale: no difficulty, some difficulty, a lot of difficulty and cannot do. Participants who reported “some or worse” difficulty with vision (with or without glasses) or hearing (with or without a hearing aid) were then asked about self-reported AD awareness, use, unmet/undermet need and barriers to access in vision and hearing domains, respectively, using relevant questions from the WHO rATA questionnaire with accompanying pictorial showcards and item descriptions (see Supplementary File S1) [5,7]. Self-reported need included both unmet need (reported not having AD but needing AD) and undermet need (reported having AD, but needing improved AD).

### 2.2. Vision Clinical Assessment

Distance and near visual acuity (VA) were measured indoors by the team optometrist or optometry technician as follows:

Distance VA: Uncorrected VA and corrected VA (wearing glasses, if available) were measured at 3 metres using Peek Acuity, a validated visual acuity test on tablet devices [16]. All participants with presenting VA (uncorrected VA or corrected VA if wearing glasses) less than 6/12 in either eye underwent (1) a pinhole test in the eye(s) less than 6/12 and (2) objective (retinoscopy) and subjective refraction of both eyes using a trial lens set and fixed wall chart. Best corrected visual acuity (BCVA) was measured with Peek Acuity following refraction. Participants with uncorrected VA < 6/12 in the better eye which improved to 6/12 or better with corrected VA, pinhole VA or BCVA were classified as “total need” having any refractive error (RE); participants who could see 6/12 or better with their own distance glasses were categorised as having “met need”; participants who could not see 6/12 with their own distance glasses but could be corrected to 6/12 or better were categorised as having “undermet need” and required updated distance glasses; and participants without glasses who could be corrected to 6/12 or better with pinhole or refraction were categorised as having “unmet need”. Uncorrected refractive error (URE) includes both “unmet need” and “undermet need”.

Near vision screening: Presenting near vision (uncorrected or wearing glasses, if available) was screened at N8 threshold. A binary outcome of can or cannot identify 4 out of 5 tumbling E optotypes at 40cm was recorded. Participants who were able to see N8 wearing near vision glasses were categorised as “met need”. Participants unable to see N8 were re-tested using an age-appropriate near add correction in trial frame and classified as needing either new near glasses (unmet need) or updated near glasses (undermet need) depending on glasses ownership.

All participants were assessed for contrast sensitivity and intraocular pressure, and a dilated clinical eye examination (eyelids, anterior and posterior segment eye disease) was undertaken.

For people with vision impairment (VI), the main cause of VI was assigned following WHO protocol of “easiest to treat” [11].

### 2.3. Hearing Clinical Assessment

In one team, the audiology nurse screened for hearing impairment (HI) using the Rapid Assessment of Hearing Loss (RAHL) methodology [12]. All participants completed a questionnaire on clinical history and risk factors for hearing loss, and then underwent a hearing test using HearTest, a validated mobile-based pure tone audiometry application [17]. Hearing was assessed in a separate, private area to minimise ambient noise levels. All participants assessed by this field team with the audiology nurse then had their ears examined by the audiology nurse using an otoscopy to assess for presence of ear diseases. Participants with hearing loss additionally underwent tympanometry. A probable cause of hearing loss was recorded (based on findings from hearing test, otoscopy, tympanometry and clinical history) and grouped in three broad categories as probable conductive, sensorineural, or mixed. For the purposes of this study, participants with bilateral sensorineural or mixed type of hearing loss (HL) (better ear > 25 dB) were categorised as likely “needing a hearing aid following diagnostic audiology review” [12].

### 2.4. Vision and Hearing Clinical Assessment Threshold Definitions

In this paper, “mild/worse VI” will be used to refer to the threshold of VA < 6/12 in the better eye, and “moderate/worse VI” will be used to refer to the threshold of VA < 6/18 in the better eye, based on WHO vision categories. For hearing, based on WHO categories, “mild/worse HI” will be used to threshold of HL > 25 dB in the better ear and “moderate/worse HI” will be used to refer to HL > 40 dB in the better ear.

### 2.5. Data Analysis

Stata 16.0 (StataCorp LP, College Station, TX, USA) was used to analyse the data. The ‘svy’ command was used to derive proportion estimates accounting for cluster sampling. Data from the 2013 population housing census were used to create weights which were then used to adjust the prevalence estimates (of impairment and need/unmet for AD) for age, sex and regional clusters for vision and age and sex only for hearing, to account for differences in the sample and census population.

We calculated AD unmet and total need, coverage and effective coverage separately for mild/worse and moderate/worse vision and hearing impairment thresholds. The exact definitions with vision and hearing thresholds are listed in table footnotes and Supplementary File S2. Broadly, we used the following definitions based on clinical assessment:

- Met need: Needs and observed to be using an appropriate AD/total population examined.
- Undermet need: Needs and observed to be using an AD which did not correct vision/hearing to required threshold/total population examined.
- Unmet need: Needs but not observed to be using the AD/total population examined.
- Total need: (summation of met need + undermet need + unmet need)/total population examined.
- Coverage: (met need + undermet need)/total need.
- Effective coverage [18] (for glasses only): met need/total need.

Socioeconomic status was calculated using the Equity tool wealth quintiles based on national scores. Estimates of “total need” for glasses (distance and near) and hearing aids were stratified by age, sex, socioeconomic status and urban/rural location with logistic regression used to calculate test for trends. These analyses were also calculated to account for the weighting and clustering. Self-reported functional limitations and unmet/undermet need for glasses and hearing aids were compared to AD unmet/undermet need identified through clinical impairment assessment, making the assumption that clinical assessment provides more reliable data (definitions in Supplementary File S2).

### 2.6. Ethical Considerations

Ethical approval was granted by The London School of Hygiene & Tropical Medicine Ethics Committee and The Gambia Government/Medical Research Council Joint Ethics Scientific Coordinating Committee (see Supplementary File S1). All participants were either given or read a participant information sheet in the participant’s respective local language which covered the risks and benefits of taking part in the study. Informed consent in the form of a signature or thumb print was obtained from all research participants. Participants identified as needing vision or hearing ADs and/or other services were referred as appropriate using the survey referral form.

## 3. Results

A total of 11,027 people were enumerated and 9188 participants underwent vision screening (response rate 83.3%). A total of 2935 people were enumerated in the clusters where hearing assessment was included and 1393 participants underwent hearing assessment (response rate 47.5%). Demographic characteristics of the 2013 census population, vision study sample and hearing study sample are presented in Supplementary File S3a. For demographic characteristic comparison specifically regarding the hearing assessment responders versus non-responders, sex and urban/rural location were fairly similar; however, slightly more older people did not respond (see Supplementary File S3b).

Overall vision and hearing clinical impairment (from all causes) and self-reported difficulty results are presented in Table 1. The prevalence of presenting distance VI mild/worse (better eye VA < 6/12, all causes) was 13.4% (95% CI 12.4–14.4, n = 1327), and the prevalence of presenting distance VI moderate/worse (better eye VA < 6/18, all causes) was 10.0% (95% CI 9.2–10.9, n = 1001). Presenting near VI prevalence was 53.4% (95% CI 51.7–55.2, n = 4774). Self-reported “some or worse” visual difficulty was 26.9% (95% CI 25.2–28.7, n = 2530) and for “a lot or worse”, it was 2.0% (1.7–2.4, n = 179).

The prevalence of mild/worse HI (>25 dB) was 28.1% (95% CI 24.6–31.9, n = 402) and moderate/worse HI (>40 dB) was 1.6% (95% CI 1.0–2.6, n = 24). Self-reported “some or worse” hearing difficulty was 1.7% (95% CI 0.9–3.2, n = 385) and for “a lot or worse”, it was 0.2% (0.04–0.5, n = 55).

### 3.1. Estimated Population AD Need and Coverage

Table 2 presents estimated unmet need, total need and coverage of each AD based upon clinical impairment assessment. Effective coverage is presented for glasses only. Population estimates of AD need are presented for people with (i) mild/worse impairment and (ii) moderate/worse impairment only based on gaps identified in previous papers and definitions used to indicate different impairment AD cut-off levels [6]. The exception is near glasses where only a binary cut-off was used.

#### 3.1.1. Distance Glasses

Overall, based on mild/worse VI, the prevalence of total need for distance glasses (i.e., any RE) was 5.6% (95% CI 5.0–6.3, n = 546) and unmet need was 5.4% (95% CI 4.8–6.0, n = 529). Only 14 people were observed as having met needs and 3 had an undermet need for glasses. Based on moderate/worse VI, the prevalence of total need for distance glasses was 4.3% (95% CI 3.8–4.9, n = 435) and unmet need was 4.3% (95% CI 3.8–4.9). Only 10 people were observed as having met need and 4 had an undermet need. Coverage was therefore low for both mild/worse VI (3.8%, 95% CI 2.3–6.3) and moderate/worse VI (3.5%, 95% CI 2.0–6.0) cut-offs, and even lower for effective coverage (mild/worse VI: 3.3%, 95% CI 1.9–5.8; moderate/worse VI: 2.7%, 95% CI 1.4–5.0).

#### 3.1.2. Near Glasses

The prevalence of total need for near glasses was 45.9% (95% CI 44.2–47.5). The prevalence of unmet near glasses need was 44.9% (95% CI 43.2–46.5, n = 3942). Only 8 people were classified as having met need and 63 as having an undermet need. There was, therefore, low coverage of near glasses (2.2%, 95% CI 1.6–3.0) and even lower effective coverage (0.2%, 95% CI 0.09–0.4).

#### 3.1.3. Hearing Aids

Overall prevalence of total need for hearing aids based on mild/worse HI was 25.5% (95% CI 22.2–29.2, n = 367), while based on moderate/worse HI, it was 1.5% (95% CI 0.9–2.4, n = 23). Only one participant was identified as wearing a hearing aid, but they were referred for further diagnostic audiology and possible hearing aid fitting. Therefore, there was no met need and no effective coverage.

#### 3.1.4. Total Need for Distance Glasses, Near Glasses and Hearing Aids by Sex, Age, Wealth Quintile and Location

The total need for distance glasses (mild/worse VI and moderate/worse VI), near glasses and hearing aids (both mild/worse HI and moderate/worse HI) all increased significantly with age (*p* < 0.01) (Table 3). The need for distance glasses (mild/worse VI and moderate/worse VI) and hearing aids (mild/worse HI) was significantly higher among women compared to men (*p* < 0.02). In contrast, the need for near glasses was significantly higher among men compared to women (*p* < 0.001). There were no differences between the socioeconomic categories or urban and rural categories. With adjustment for age and sex, these results were essentially unchanged.

### 3.2. Self-Reported AD Awareness, Need and Access Barriers

Table 4 presents self-reported awareness, unmet/undermet need and barriers to accessing vision and hearing ADs out of the participants who self-reported “some or worse” difficulty in vision and hearing. As presented in Table 1, for vision, 2530 (26.9%, 95% CI 25.2–28.7) participants self-reported “some or worse” difficulty seeing either with or without glasses, and, for hearing, 385 (1.7%, 95% CI 0.9–3.2) participants reported “some or worse” difficulty in hearing either with or without hearing aids.

#### 3.2.1. Vision ADs, Including Glasses

Of those who self-reported “some or worse” difficulty in vision (n = 2530), 72.8% (n = 1816) reported awareness of spectacles and 8.3% (n = 209) reported awareness of white canes, while awareness was low (<4%) for other vision ADs including talking or touching watch, magnifier or telescope, and braille equipment. Overall, 28.0% (n = 709) reported being unaware of any vision AD. In terms of self-reported unmet/undermet need, 66.4% (n = 1681) reported an unmet/undermet need for spectacles, while <1% reported needing each of the other vision ADs. Overall, 17.7% (n = 447) reported not needing any vision AD. Of those who reported unmet/undermet need for spectacles, the most commonly reported access barriers were AD not locally available (44%), transport not available (43%), and cannot afford (35%).

#### 3.2.2. Hearing ADs, Including Hearing Aids

Of those who self-reported “some or worse” difficulty in hearing (n = 385), 86.0% (n = 331) were not aware of any hearing AD. Overall, 12.5% (n = 48) reported prior awareness of hearing aids, 2.1% (n = 8) reported awareness of alarm signallers with light/vibration and <1% (n = 2) reported awareness of personal frequency modulation (FM) system. For self-reported unmet/undermet need, 58.0% (n = 223) reported needing hearing aids, 8.8% (n = 34) needing alarm signallers and 2.6% (n = 10) needing personal FM system. Just over a third (34.7%; n = 140) reported not needing any hearing AD. Of those who reported unmet/undermet need for hearing aids, most common access barriers were AD not locally available (76%), transport not available (74%), unaware of AD (62%) and no one available to instruct how to use (59%).

### 3.3. Relationship between AD Need Measurement Approaches

**Figure 1:**
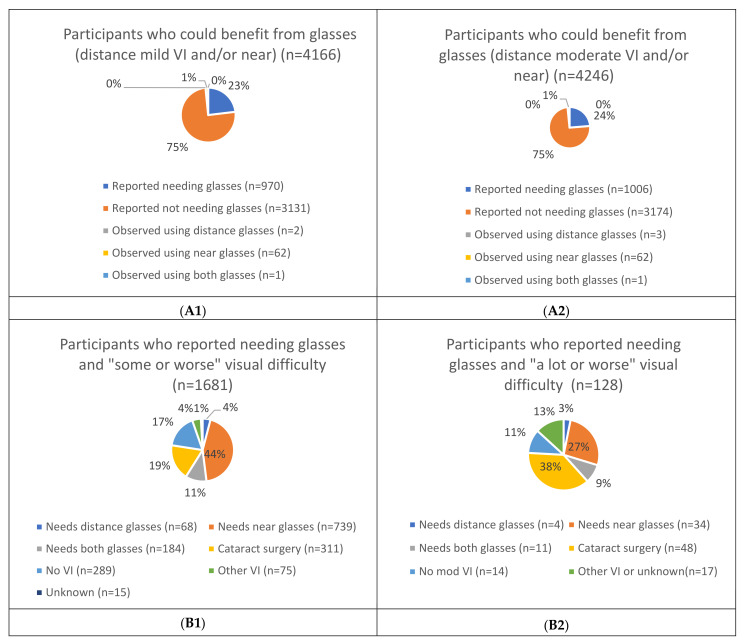
Comparing reported versus clinical impairment assessment unmet/undermet need for near and/or distance glasses (mild vs. moderate vision impairment (VI)). (**A1**)**:** Participants who could benefit from glasses (distance mild VI and/or near) (n = 4166), (**A2**): Participants who could benefit from glasses (distance moderate VI and/or near) (n = 4246), (**B1**): Participants who reported needing glasses and “some or worse” visual difficulty (n = 1681), (**B2**): Participants who reported needing glasses and “a lot or worse” visual worse difficulty (n = 128).

**Figure 2:**
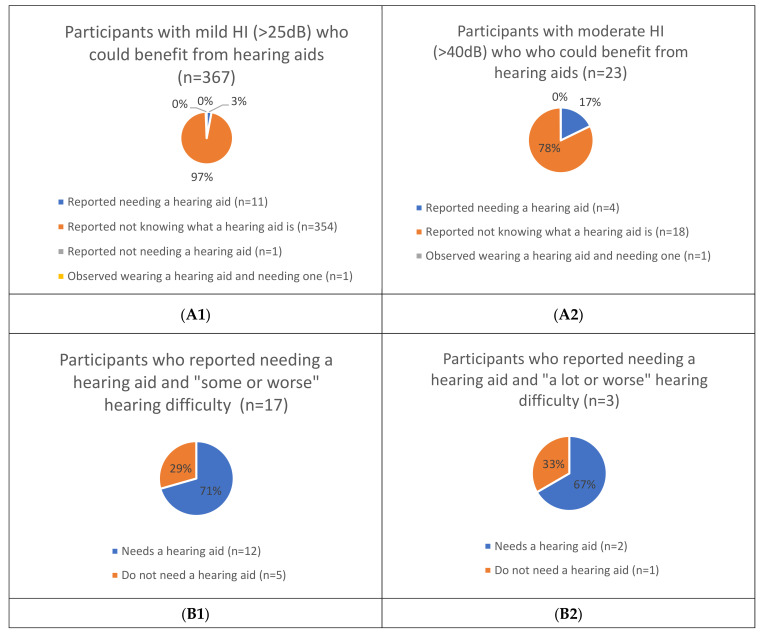
Comparing reported versus clinical impairment assessment unmet/undermet need for hearing aids for both mild and moderate hearing impairment (HI). (**A1**): Participants with mild HI (>25 dB) who could benefit from hearing aids (n = 367), (**A2**): Participants with moderate HI (>40 dB) who could benefit from hearing aids (n = 23), (**B1**): Participants who reported needing a hearing aid with “some or worse” hearing difficulty (n = 17), (**B2**): Participants who reported needing a hearing aid with “a lot or worse” hearing difficulty (n = 3).

Figure 1 and Figure 2 present the relationship between the two different approaches for assessing near and distance glasses’ (mild/worse and moderate/worse VI) and hearing aids’ (mild/worse and moderate/worse HI) unmet/undermet need (see Supplementary File S4).

#### 3.3.1. Self-Reported Unmet/Undermet Need for Glasses (Distance and/or Near) among People with Near Vision and/or Distance (Mild/Worse and Moderate/Worse VI) Uncorrected Refractive Error

Of the 4166 people identified as having near and/or distance (mild/worse VI) URE, three-quarters (75%, n = 3131) self-reported they did not need glasses (see Figure 1(A1)). Similarly, of the 4246 people identified as having near and/or distance (moderate/worse VI) URE, 3174 (75%) reported not needing distance glasses (see Figure 1(A2)).

Of the 1681 people who self-reported “some or worse” visual difficulty and needing glasses, 991 (60%) actually needed near and/or distance glasses (for mild/worse VI) based on clinical impairment assessment. Of the remaining participants, 311 (19%) needed cataract surgery, 75 (4%) had other causes of VI where glasses would not be of benefit, 15 (1%) had an unknown cause and 289 (17%) did not have VI (see Figure 1(B1)). Of the 128 people who self-reported “a lot or worse” visual difficulty and needing distance glasses only, 49 (38%) actually needed near and/or distance glasses (moderate/worse VI) based on clinical assessment. Of the remaining participants, 48 (38%) needed cataract surgery, 17 (13%) had other or unknown causes of VI and 14 (11%) did not have moderate/worse VI (see Figure 1(B2)).

#### 3.3.2. Self-Reported Unmet/Undermet Need for Hearing Aids among People with Mild/Worse HI (>25 dB) and Moderate/Worse HI (>40 dB)

Of the 367 people with mild/worse HI (>25 dB) who were clinically assessed to likely need hearing aids, 11 (3%) reported needing one, 354 (97%) reported not knowing what it was and <1% (2 participants) were either observed to be wearing one (n = 1) or reported not needing one (n = 1) (see Figure 2(A1)). Of the 23 people with moderate/worse HI (>40 dB) who were clinically assessed to likely need hearing aids, 4 (17%) reported needing one, 18 (78%) reported not knowing what it was, and 1 (<1%) was observed to be wearing one (see Figure 2(A2)).

Of the 17 people who self-reported needing hearing aids with “some or worse” hearing difficulty, 12 (71%) actually needed hearing aids based on clinical assessment and 5 (29%) did not (see Figure 2(B1)). Of the three people who self-reported needing hearing aids with “a lot or worse” hearing difficulty, two (67%) actually needed hearing aids based on clinical assessment and one (33%) did not (see Figure 2(B2)).

## 4. Discussion

### 4.1. Estimated Population AD Need and Coverage

This study found evidence of high need and very low coverage of two priority ADs (glasses and hearing aids) among adults aged 35+ years in The Gambia based on clinical impairment assessment. Total need was highest for near glasses (45.9%), followed by hearing aids based on mild/worse HI (25.5%), and distance glasses was lower (mild/worse VI: 5.6%; moderate/worse VI: 4.3%). The total need for all ADs increased significantly with age. Total need was significantly higher among females compared to males for distance glasses (mild/worse VI and moderate/worse VI) and hearing aids (mild/worse HI), and significantly higher among males compared to females for near glasses.

AD coverage was very low with fewer than 4% of people who needed distance glasses, near glasses or hearing aids actually observed wearing them. These findings further indicate that glasses and hearing aid provision services are very limited in The Gambia and need to be scaled up [2]. For example, for vision, the initiative One Sight worked with The Gambian government to support the development of seven vision centres and job creation, but the network of services requires further expansion [19]. For hearing, though some health facilities provide basic ear, nose and throat (ENT) services to treat minor cases, all major cases and anyone with hearing difficulties in the entire country are referred to the Polyclinic to the one Audiology nurse for hearing assessments and hearing aid fittings in collaboration with St Johns School for the Deaf in the capital city Banjul. A lack of audiology service provision is congruent with the findings of a survey conducted to determine the current status of ENT, audiology, and speech therapy services between 2009 and 2015 in 15 sub-Saharan African countries [20]. Human resources especially need to be scaled up so more ENT doctors, audiology nurses and speech therapists are trained and available to provide hearing health services in The Gambia.

Compared to other studies of multi-domain clinically assessed AD need, our findings align with a survey in Cameroon showing high need and low coverage for distance glasses and hearing aids [6]. A survey in India found similarly low coverage of hearing aids, but much higher coverage of distance glasses both for mild/worse VI at 44% and moderate/worse VI at 87% which might indicate a greater access to vision services in that setting [6].

For vision, it is challenging to compare our glasses (distance and near) unmet need results with the previous 1996 Gambia National Eye Health study, given differing definitions and methods of calculating these estimates [21]. Our finding for distance glasses unmet need (5.4%) was slightly lower than the 7.5% estimate reported in a 15+ years old Tanzanian population study, despite the lower population age range, although spectacle coverage was similarly low (distance glasses: 1.69% and near vision glasses: 0.42%) [22]. Our estimate of unmet need for near vision glasses (44.9%) was similar when compared to this same Tanzanian study’s population aged 35+ years which found an uncorrected presbyopia prevalence of 46.5% [22] and slightly lower than studies in similar age groups in Ghana (64%) [23] and Nepal (66.1%) [24]. For hearing, the need for hearing aids (25.5%) in this study is lower than estimates from RAHL surveys, which used the same ear/hearing assessment methods, in Malawi (30.8%) [25] and China (54%) [26], likely because the focus was people 35+ compared to 50+ in the other surveys. The low coverage (<1%) is similar across all three studies [25,26].

However, it is also noted that population-based clinical impairment studies often do not explicitly measure or present specific AD need alongside estimates of impairment type, cause and severity. Therefore, to address the AD data gap, it is recommended that future surveys include these estimates on AD need and coverage for vision and hearing as well as the assessment of additional functional domains and related ADs. Additionally, this paper provides comparison between mild and moderate impairments for distance glasses and hearing aids. Though unmet need for moderate/worse VI/HI is more imperative, our analyses show that a high number of people with mild VI and HI might also benefit functionally from distance glasses and hearing aids. It is recommended that the mild/worse threshold is reported as need for these two ADs at a minimum, which is congruent with recent recommendations [18] and will have programme implications given the increase in needs identified.

### 4.2. Self-Reported AD Awareness, Need and Access Barriers

Our findings further emphasise that glasses are the most well-known vision AD (72.8%), and that the use of pictorial showcards with item descriptions appears to enhance understanding of the ADs in participants (i.e., for hearing aids among participants who reported “some or more” difficulty hearing, 12.5% were initially aware of this AD compared to 58.0% who reported unmet/undermet need after learning about this AD), so this is recommended in future self-reported studies [5]. Additionally, the self-reported low AD awareness and unmet/undermet need results are congruent with our low glasses and hearing aid coverage findings, and further reinforce the need for increased AD advocacy, awareness raising activities and service provision. Further, when using the cut-off of “some or worse” difficulty in seeing or hearing, it is possible that participants who reported no problem seeing/hearing were aware of the ADs and/or might self-report still needing ADs; therefore, they might have been missed in our findings. It will be important for future research to explore the accuracy of this cut-off to determine AD need. Finally, there are anecdotal reports of societal stigma associated with use of certain AD such as glasses, especially among younger people. Such views could be explored through qualitative research to explore attitudes and stigma towards ADs that may adversely affect the widespread utilization of ADs.

### 4.3. Relationship between AD Need Measurement Approaches

The agreement between AD unmet/undermet need measured by self-report and by clinical impairment assessment, for glasses (near and distance) and hearing aids, was very poor. Additionally, awareness about ADs was low, even with the addition of AD pictures and item descriptions during the survey. Further, AD unmet/undermet need was consistently either under-reported or over-reported. For example, hearing aids were under-reported by people who were clinically assessed to need them given poor awareness of what they were, and 75% of people who were clinically assessed to need near and/or distance (mild/worse VI) glasses did not perceive any need for glasses. At the same time, AD need was over-reported, given at least 40% of those who self-reported needing glasses (distance and/or near) actually did not need them. Our findings are similar to previous findings from the Cameroon and India AD study, even with additional breakdown by level of severity for distance and hearing aids and two types of glasses (distance and near) [6]. Though self-report is a quicker and lower cost method, our findings suggest that when self-report is solely used, estimates will likely be inaccurate, given both the overestimations and underestimations of need.

Research comparing AD need measurement approaches is limited as population surveys typically use either self-reported AD methodology or clinical impairment assessment methodology. For example, Pryor et al.’s study in two districts in Bangladesh solely used WHO GATE’s rATA [5] and SINTEF’s multiple population-based surveys present self-reported AD need only, often in category groupings by functional domain and/or type [27,28,29,30,31,32,33,34,35].

It is also important to note that our comparative findings rely on clinical impairment assessments as the “gold standard”, and there are limitations with this approach as well. Most notably, impairment assessment methods typically only focus on the more “medical” component of body structure and function in the international classification of functioning, health and disability (ICF) [36]. In order to measure AD need, more comprehensive data are required to be collected about the person’s broader functional needs, incorporating the other ICF components including his/her activities, participation, and personal and environmental factors [6] and may explain some of the disconnect in the data. For example, an individual who is clinically identified to need distance (mild/worse VI) and near glasses living in a rural area of The Gambia who does not drive and is not literate may not perceive his/her visual functioning as a problem, given it does not appear to impact on life activities and/or participation. Additionally, an individual who is illiterate may prefer to obtain their information from auditory sources, such as radio and/or word of mouth, and may not have a need for reading glasses to read a newspaper. Therefore, gathering more of an understanding about what is needed to support activity participation, contextual factors, any necessary social and/or environmental accommodations are essential towards contributing to AD assessment. This further emphasises the need to support the development of hybrid methodology integrating self-report, clinical impairment and functional assessment methodologies. This proposed comprehensive method to determine AD need is important to inform policy-driven efforts towards achieving Sustainable Development Goal 3 and Universal Health Coverage.

### 4.4. Study Strength and Limitations

This study provided population-based estimates for three ADs in The Gambia based upon standardised clinical impairment assessment procedures. It also included self-reported AD measures which enabled comparison of the two measurement approaches, including different severity levels. Further, uncorrected VA was measured in addition to presenting which provided more accurate data for met and undermet need, as well as for coverage and effective coverage than using methods that aligned with the recent vision sector indicator [18].

However, there were limitations. There were higher than expected incomplete examinations and non-response rates, and an under-sampling of younger men due to the pre-rainy and rainy/farming season skewing the survey sample towards females, which required sampling weights. Logistical challenges, such as finding adequate space for the central location set up in each cluster, often resulted in delays, and human resource constraints did not allow for continuity of examiners during the survey which potentially led to measurement bias. It was also challenging to find quieter areas for conducting the hearing test and the hearing survey response rate was low (47.5%). This may be related to response burden as the hearing assessment was often conducted at the end of all the other survey assessments [11]. It is important for future multiple functional domain surveys to consider order of assessments and length of data collection. Additionally, it is possible that the unmet need for hearing aids is overestimated, given this estimate is based upon possible cause diagnoses of mixed or sensorineural hearing loss which indicates diagnostic audiology services and *possible* hearing aids fittings, due to difficulties accurately assessing this in the field.

It is noted that AD ‘use’ was reported in three different sections of the survey by different data collector cadres, and our analysis was based on participants being observed wearing the AD at the time of the clinical assessment conducted at a central point. However, this may have led to underestimates of use as participants might have been unaware they should bring their glasses/hearings aids or expected a new pair of glasses or hearing aid following the exam. It is recommended that future surveys provide clear communication indicating if the AD should be worn at the central location and if there will or will not be provision of free ADs following a survey. Further, for the self-report data, participants were only asked about ‘glasses’ overall, not separately for distance and near glasses, which limited our comparisons with the clinically assessed glasses need. Given the large difference in need, it is recommended that future surveys using self-report (e.g., rATA) should ask about need for distance and near glasses separately.

Finally, this study explored need in an adult 35+ population for only 2 of the 50 priority ADs. Future studies are needed to assess access for younger age groups, and data on other ADs are lacking and should be included in future data collection efforts when possible. For example, low vision AD need should also be assessed in VI surveys. Specifically, as part of the broader survey, contrast sensitivity impairment was clinically measured; however, it was not fully assessed with regard to AD need [11]. It is recommended in future vision surveys that this, alongside other vision AD needs, are explored and assessed further, including exploring appropriate cut-off points for the AD required, such as for filter glasses with contrast sensitivity. A recommendation to address the data gap is to work with the GBoS to incorporate AT assessment tools into the Demographic and Health Surveillance (DHS), which are conducted every three years, and the next national population census scheduled for 2023.

## 5. Conclusions

In The Gambia, the need for distance glasses, near glasses and hearing aids is high, yet coverage is very low. Our findings generated much-needed data on population-based AD need in this setting. It will be important for national health, local policy and social services planning to address the barriers identified to accessing ADs, whilst supporting the essential development of vision and hearing AD services, including rehabilitation, with the overall aim to improve functioning and quality of life for individuals in The Gambia. Additionally, our methodological comparison highlights limitations when using self-report alone and further emphasises the need for improved population-level survey methods to estimate AD need. Scheduled GBoS surveys are opportunities that can incorporate AT assessment tools to address this data gap in The Gambia.

## Figures and Tables

**Table 1:** Overall prevalence of vision and hearing impairment/difficulty with participants 35+ who completed distance vision, near vision and hearing clinical assessments and self-reported difficulty questioning in The Gambia.

Functional Domain	Total Number Participants		Prevalence ^ % (95% CI)
	Assessed (N)	With Impairment/Difficulty (N)	
VISION			
Vision clinically assessed			
Distance vision			
Mild or worse (VA < 6/12)	9188	1327	13.4 (12.4–14.4)
Moderate or worse (VA < 6/18)	9188	1001	10.0 (9.2–10.9)
Near vision*	9183	4774	53.4 (51.7–55.2)
Self-reported vision difficulties **			
“Some or worse” difficulty	9180	2530	26.9 (25.2–28.7)
“A lot of or worse” difficulty	9180	179	2.0 (1.7–2.4)
HEARING			
Hearing clinically assessed			
Mild or worse (>25 dB)	1393	402	28.1 (24.6–31.9)
Moderate or worse (>40 dB)	1393	24	1.6 (1.0–2.6)
Self-reported hearing difficulties ***			
“Some or worse” difficulty	9185	385	1.7 (0.9–3.2)
“A lot of or worse” difficulty	9185	55	0.2 (0.04–0.5)

^ Crude counts with prevalence adjusted for cluster, age and sex for vision, and adjusted for age and sex for hearing; * Test not possible with 5 participants; ** 8 participants were missing Washington group data, n = total participants who self-reported “some or worse” difficulty seeing either with or without glasses; *** 3 participants were missing Washington group data, n = total participants who self-reported “some or worse” difficulty hearing either with or without hearing aids.

**Table 2:** Three assistive devices total need, unmet need, coverage and effective coverage estimates in The Gambia ^^.

Assistive Devices	Total Need ^^,^*		Unmet Need ^^,^**		Coverage ^^,^***	Effective Coverage ^^,^****
	N	% (95% CI)	N	% (95% CI)	% (95% CI)	% (95% CI)
Distance glasses (mild/worse VI)	546	5.6 (5.0–6.3)	529	5.4 (4.8–6.0)	3.8% (2.3–6.3)	3.3% (1.9–5.8)
Distance glasses (moderate/worse VI)	435	4.3 (3.8–4.9)	421	4.2 (3.6–4.7)	3.5% (2.0–6.0)	2.7% (1.4–5.0)
Near glasses ^&^	4013	45.9 (44.2–47.5)	3942	44.9 (43.2–46.5)	2.2% (1.6–3.0)	0.2% (0.09–0.4)
Hearing aids (mild/worse HI)	367	25.5 (22.2–29.2)	366	25.5 (22.1–29.2)	0.1% (0.02–1.0)	-
Hearing aids (moderate/worse HI)	23	1.5 (0.9–2.4)	22	1.5 (0.9–2.4)	2.3% (0.3–15.9)	-

Abbreviations: VI = vision impairment, HI = hearing impairment, CI = confidence interval; ^ Crude counts with prevalence adjusted for cluster, age and sex for vision, and adjusted for age and sex for hearing; ^^ vision total population n = 9188, hearing total population n = 1393; ^&^ Near glasses missing data on 12 participants; * total need = (met need + undermet need + unmet need)/total population, see manuscript’s Methods and Results sections and Supplementary File S2 for details; ** unmet need: for distance glasses (mild/worse VI), unmet need = participants without glasses who could be corrected to 6/12 or better with pinhole or refraction; for distance glasses (moderate/worse VI), unmet need = participants without glasses who could be corrected to 6/18 or better with pinhole or refraction; for near glasses, unmet need = participants with distance BCVA of ≥6/12 in at least one eye who do not have correction for near and whose near PVA is <N8 but can be corrected to N8; for hearing aid (mild/worse HI), unmet need = referred to diagnostic audiology and possible hearing aid due to bilateral sensorineural or mixed type of hearing loss (better ear >25dB) causes; for hearing aid (moderate/worse HI), unmet need = referred to diagnostic audiology and possible hearing aid due to bilateral sensorineural or mixed type of hearing loss (better ear >40 dB) cause; *** coverage = (met need + undermet need)/total need; **** effective coverage (for glasses only) = met need/total need.

**Table 3:** Total need for distance glasses, near glasses, and hearing aids stratified by sex, age, socioeconomic status and urban/rural in The Gambia.

	Distance Glasses				Near Glasses ^		Hearing Aids			
	<6/12 (Mild/Worse VI) ^		<6/18 (Mod/Worse VI) ^				>25 dB (Mild/Worse HI)^		>40 dB (Mod/Worse HI) ^	
	N	% (95% CI)	N	% (95% CI)	N	% (95% CI)	N	% (95% CI)	N	% (95% CI)
**Sex**										
Male	178	5.0 (4.2–5.8)	128	3.4 (2.7–4.2)	1440	48.3 (47.5–50.9)	113	20.6 (16.5–25.3)	8	1.3 (0.5–3.1)
Female	368	6.3 (5.6–7.1)	307	5.2 (4.5–5.9)	2573	43.4 (41.8–44.9)	254	30.7 (26.6–35.0)	15	1.7 (1.0–3.1)
*p*-value	**0.01**		**<0.001**		**<0.001**		**<0.001**		0.61	
**Age groups**										
35 to <50 years	101	2.1 (1.6–2.8)	73	1.4 (1.0–2.0)	1283	29.5 (27.5–31.6)	119	12.6 (9.8–16.1)	9	0.8 (0.4–1.9)
50 to <60	114	6.3 (5.2–7.7)	90	4.8 (3.8–6.1)	1240	76.3 (73.4–78.9)	82	29.1 (23.5–35.3)	1	0.3 (0.04–2.4)
60+ years	331	14.0 (12.5–15.6)	272	11.2 (9.8–12.8)	1490	62.2 (59.7–64.6)	166	56.9 (49.5–63.9)	13	4.3 (2.3–8.1)
*p*-value	**<0.001**		**<0.001**		**<0.001**		**<0.001**		**0.006**	
Socioeconomic status *										
1st quintile	49	5.4 (3.9–7.4)	32	3.4 (2.2–5.2)	353	44.1 (39.0–49.2)	28	22.7 (13.3–36.0)	0	-
2nd quintile	72	5.5 (3.9–7.6)	59	4.4 (3.0–6.5)	535	43.9 (40.2–47.7)	63	30.8 (23.6–39.0)	4	1.4 (0.5–3.5)
3rd quintile	122	5.0 (4.0–6.1)	102	4.1 (3.2–5.1)	973	45.0 (41.7–48.5)	78	26.2 (21.0–32.1)	7	2.4 (0.9–6.4)
4th quintile	128	5.9 (4.8–7.1)	113	4.9 (3.9–6.1)	927	46.6 (43.4–49.7)	93	23.6 (18.3–29.8)	5	0.9 (0.4–2.1)
5th quintile	175	6.2 (5.1–7.4)	129	4.3 (3.5–5.2)	1225	47.7 (45.4–49.9)	105	25.3 (19.7–31.7)	7	2.1 (1.0–4.3)
*p*-value	0.31		0.46		0.08		0.67		0.31	
**Location**										
Urban	309	5.8 (5.0–6.7)	250	4.5 (3.8–5.2)	2259	47.3 (45.4–49.2)	222	24.1 (20.1–28.6)	14	1.6 (1.0–2.8)
Rural	237	5.4 (4.5–6.4)	185	4.1 (3.2–5.1)	1754	44.1 (41.3–47.0)	145	27.9 (22.3–34.3)	9	1.3 (0.5–3.4)
*p*-value	0.54		0.49		0.07		0.30		0.66	

Abbreviations: VI = vision impairment, HI = hearing impairment, Mod = moderate, CI = confidence interval. ^ Crude counts and prevalence adjusted for cluster, age and sex weighting for vision, and adjusted age and sex weighting for hearing; the CIs presented are calculated using standard errors that account for the effect of weighting and clustering for vision only, logistic regression was used to calculate test for trends; * Equity Tool quintile based on national scores.

**Table 4:** Self-reported awareness, need and barriers to accessing vision and hearing assistive devices amongst participants who self-reported “some or worse” difficulty in vision and hearing, respectively.

Assistive Devices by Domain	VISION							HEARING				
	Spectacles	Talking or Touching Watch	Magnifier or Telescope	White Cane	Braille Equip-ment	Other	None	Alarm Signallers ^	Hearing Aids and Batteries	Personal Frequency Modulation System	Other	None
Awareness of AD *	1816 (72.8%)	37 (1.5%)	84 (3.3%)	209 (8.3%)	62 (2.5%)		709 (28.0%)	8 (2.1%)	48 (12.5%)	2 (<1%)		331 (86.0%)
Unmet/undermet need *	1681 (66.4%)	12 (<1%)	7 (<1%)	17 (<1%)	1 (<1%)	8 (<1%)	447 (17.7%)	34 (8.8%)	223 (58.0%)	10 (2.6%)	3 (<1%)	140 (36.4%)
**Barriers to not having AD ^**												
Unaware of AD	544 (32%)	5 (42%)	2 (29%)	8 (47%)	-	4 (50%)		27 (79%)	138 (62%)	7 (70%)	33%	
AP not locally available	741 (44%)	4 (33%)	3 (43%)	8 (47%)	1 (100%)	5 (63%)		6 (18%)	170 (76%)	9 (90%)	33%	
Cannot afford	581 (35%)	5 (42%)	2 (29%)	12 (71%)	1 (100%)	4 (50%)		5 (15%)	122 (55%)	6 (60%)	33%	
Not suitable for home/environment	102 (6%)	-	1 (14%)	1 (6%)	-	2 (25%)		2 (6%)	21 (9%)	1 (10%)	-	
No one available to instruct how to use	567 (34%)	5 (42%)	3 (43%)	6 (35%)	1 (100%)	2 (25%)		5 (15%)	131 (59%)	6 (60%)	33%	
Transport not available	716 (43%)	5 (42%)	3 (43%)	8 (47%)	1 (100%)	4 (50%)		4 (12%)	166 (74%)	8 (80%)	66%	
Do not like appearance	15 (1%)	-	-	-	-	1 (13%)		1 (3%)	4 (2%)	-	-	
People treat users differently	154 (9%)	3 (25%)	1 (14%)	6 (35%)	-	2 (25%)		4 (12%)	40 (18%)	4 (40%)	66%	
Other	47 (3%)	3 (25%)	1 (14%)	5 (24%)	-	2 (25%)		2 (6%)	9 (4%)	3 (30%)	66%	

Abbreviations: AD = assistive device; ^ Alarm signaller is with light/vibration; * for vision: Washington group data were missing for 8 participants, questions were asked to n = 2530 participants who reported “some or worse” difficulty seeing with or without glasses; for hearing: Washington group data were missing for 3 participants, questions were asked to n = 385 participants who reported “some or worse” difficulty hearing with or without hearing aids; ^ Participants could indicate ≥1 barrier selecting as many as applied; unmet/undermet need for specific AD is the denominator used for proportion.

## Data Availability

The data presented in this study are still undergoing analysis and will be made openly available once complete. The extended data presented in this study are openly available in the Open Science Framework: Gambia National Eye Health Survey 2019 Study Documents at https://doi.org/10.17605/OSF.IO/EKCDT, reference number 17 (accessed on 24 February 2021). Data are available under the terms of the Creative Commons Attribution 4.0 International license (CC-BY 4.0).

## Supplementary Materials

The following are available online at https://www.mdpi.com/article/10.3390/ijerph18126302/s1, File S1: Open Science Framework: Gambia National Eye Health Survey 2019 Study Documents, all the study materials (questionnaires, information and consent forms, show cards, etc.) are available online at https://osf.io/ekcdt (accessed on 24 February 2021). File S2: Impairment and assistive device (AD) proportion definitions using WHO vision and hearing impairment definitions. File S3, S3a: Demographic characteristics of the 2013 census population, vision study sample and hearing study sample, S3b: Demographic characteristics of the hearing survey responders and non-responders. File S4, S4a: Clinical impairment assessment and self-reported unmet/undermet need for near and/or distance glasses (both mild and moderate vision impairment), S4b: Clinical impairment assessment and self-reported unmet/undermet need for hearing aids (both mild and moderate hearing impairment).
