# Supplementary material for: Estimating Need for Glasses and Hearing Aids in The Gambia: Results from a National Survey and Comparison of Clinical Impairment and Self-Report Assessment Approaches
Source: Int J Environ Res Public Health. 2021 Jun 10;18(12):6302. doi: 10.3390/ijerph18126302 (PMC8296105; doi:10.3390/ijerph18126302)
Supplement: Supplementary file 1 [file ijerph-18-06302-s001.zip › Supplementary File S2 - FINAL.pdf]

File S2: Impairment and assistive device (AD) proportion definitions using WHO vision and hearing impairment definitions.

AD PROPORTIONS	DEFINITION
**AD need and coverage through clinical impairment assessment**	
Distance glasses for mild vision impairment (VI)	
Mild VI	Prevalence of participants with presenting visual acuity (VA) <6/12 in the better eye out of total population clinically assessed for VI.
Met need	Prevalence of participants who were observed to be wearing glasses and whose uncorrected VA (UCVA) <6/12 improved to 6/12 or better with presenting VA (PVA) in the better eye wearing their glasses.
Undermet need	Prevalence of participants who were observed to be wearing glasses and whose UCVA <6/12 did not improve to 6/12 or better with PVA in the better eye wearing their glasses but improved to 6/12 or better with pinhole or refraction.
Unmet need	Prevalence of participants who were not observed to be wearing glasses and whose UCVA <6/12 improved to 6/12 or better with pinhole or refraction.
Total need	Prevalence of summation of mild VI participants who meet 'met need', 'undermet need' or 'unmet need' definitions out of total population clinically assessed for VI.
Coverage	Proportion participants who meet 'met need' or 'undermet need' definitions out of 'total need' participants.
Effective coverage	Proportion participants who meet 'met need' definition out of 'total need' participants.
Distance glasses for moderate VI	
Moderate VI	Prevalence of participants with PVA <6/18 in the better eye out of total population clinically assessed for VI.
Met need	Prevalence of participants who were observed to be wearing glasses and whose UCVA <6/18 improved to 6/18 or better with PVA in the better eye wearing their glasses out of 'total need' participants.
Undermet need	Prevalence of participants who were observed to be wearing glasses and whose UCVA <6/18 did not improve to 6/18 or better with PVA in the better eye wearing their glasses but improved to 6/18 or better with pinhole or refraction.
Unmet need	Prevalence of participants who were not observed to be wearing glasses and whose UCVA <6/18 improved to 6/18 or better with pinhole VA or refraction in the better eye out of total population clinically assessed for VI.
Total need	Prevalence of summation of moderate VI participants who meet 'met need', 'undermet need' or 'unmet need' definitions out of total population clinically assessed for VI.
Coverage	Proportion of participants who meet 'met need' or 'undermet need' definitions out of 'total need' participants.
Effective coverage	Proportion of participants who meet 'met need' definition out of 'total need' participants.
Near glasses	
Presenting near VI	Prevalence of participants who near PVA is less than N8 at 40cm out of the total population clinically assessed for near VI.
Met need	Prevalence of participants who are observed wearing near glasses and whose near PVA is at least N8 at 40cm out of the 'total need' participants.
Undermet need	Prevalence of participants with distance BCVA of ≥6/12 in at least one eye who present with near glasses and whose PVA was <N8 but can be corrected to N8
Unmet need	Prevalence of participants with distance BCVA of ≥6/12 in at least one eye who do not have correction for near and whose near PVA is <N8 but can be corrected to N8

Total need	Prevalence of summation of near VI participants who meet 'met need', 'undermet need' or 'unmet need' definitions out of total population clinically assessed for near VI.
Coverage	Proportion of participants who meet 'met need' or 'undermet need' definitions out of the 'total need' participants.
Effective coverage	Proportion of participants who meet 'met need' definition out of the 'total need' participants.
Hearing aids for mild hearing impairment (HI)	
Mild HI	Prevalence of the participants with mild or worse hearing loss (HL) (>25dB) in the better ear out of the total population clinically assessed for HI.
Met need	Prevalence of the mild HI participants who were observed to be wearing a hearing aid and tested to have mild or worse HL (>25dB) in the better ear and bilateral sensorineural or mixed type of HL causes who were not referred to diagnostic audiology and possible hearing aid fitting out of the total need participants.
Undermet need*	Prevalence of the mild HI participants who were observed to be wearing a hearing aid and tested to have mild or worse HL (>25dB) in the better ear and bilateral sensorineural or mixed type of HL causes who were referred to diagnostic audiology and possible hearing aid fitting out of the total need participants.
Unmet need	Prevalence of participants who tested to have mild or worse HL (>25dB) in the better ear and bilateral sensorineural or mixed type of HL causes who were referred to diagnostic audiology and possible hearing aid fitting out of the total population clinically assessed for HI.
Total need	Prevalence of the summation of participants who meet 'met need', 'undermet need' or 'unmet need' definitions out of the total population clinically assessed for HI.
Coverage	Proportion of participants who meet 'met need' or undermet need' definitions out of the 'total need' participants.
Hearing aids for moderate HI	
Moderate HI	Prevalence of the participants with moderate or worse HL (>40dB) in the better ear out of the total population clinically assessed for HI.
Met need	Prevalence of the moderate HI participants who were observed to be wearing a hearing aid and tested to have moderate or worse HL (>40dB) in the better ear and bilateral sensorineural or mixed type of HL causes who were not referred to diagnostic audiology and possible hearing aid fitting out of the total need participants.
Undermet need*	Prevalence of the moderate HI participants who were observed to be wearing a hearing aid and tested to have moderate or worse HL (>40dB) in the better ear and bilateral sensorineural or mixed type of HL causes who were referred to diagnostic audiology and possible hearing aid fitting out of the total need participants.
Unmet need	Prevalence of the participants who tested to have moderate or worse HL (>40dB) in the better ear and bilateral sensorineural or mixed type of HL causes who were referred to diagnostic audiology and possible hearing aid fitting out of the total population clinically assessed for HI.
Total need	Prevalence of the summation of moderate HI participants who meet 'met need', 'undermet need' or 'unmet need' definitions out of total population clinically assessed for HI.
Coverage	Proportion of moderate HI participants who meet who meet 'met need' or undermet need' definitions out of 'total need' participants.
**Self-Reported AD Awareness, Need and Access Barriers**	
Vision	
Awareness	Proportion of participants who self-reported "some or worse" difficulty seeing (with or without glasses) who reported being aware of the vision AD.
Unmet/undermet need	Proportion of participants who self-reported "some or worse" difficulty seeing (with or without glasses) who reported needing the vision AD.

Access barriers	Proportion of specific access barrier identified out of the total number of participants who self-reported needing the vision AD with "some or worse" difficulty seeing (with or without glasses).
Hearing	
Awareness	Proportion of participants who self-reported "some or worse" difficulty hearing with or without hearing aids who reported being aware of the hearing AD.
Unmet/undermet need	Proportion of participants who self-reported "some or worse" difficulty hearing (with or without hearing aids) who reported needing the hearing AD.
Access barriers	Proportion of specific access barrier identified out of the total number of participants who self-reported needing the vision AD with "some or worse" difficulty hearing (with or without hearing aids).
**AD need proportions exploring the relationship between self-report and clinical impairment assessment**	
1. Proportion of participants identified as needing an AD through clinical impairment assessment (e.g. glasses or hearing aids) who reported "some or worse" or "a lot or worse" difficulty with seeing or hearing (with or without glasses or hearing aid respectively) using modified Washington group short set of questions. This was to assess how many people who could benefit from an AD would be captured through using self-report of "some or worse" or "a lot or worse" difficulty alone (i.e. no clinical assessment).	
2. Proportion of participants identified as needing glasses or hearing aids through clinical impairment assessment who also self-reported a need for the corresponding AD.	
3. Among people who self-reported needing glasses, the distribution of the type of glasses (e.g. distance glasses, near glasses or both distance and near glasses) and the causes of vision loss identified through clinical impairment assessment.	

* The Authors acknowledge that hearing tests cannot be completed with hearing aids on, however if a participant was observed to be wearing a hearing aid and was also referred to diagnostic audiology and possible hearing aid fitting the participant was then was listed as 'undermet need' in this analysis.
