# Supplementary material for: Estimating Need for Glasses and Hearing Aids in The Gambia: Results from a National Survey and Comparison of Clinical Impairment and Self-Report Assessment Approaches
Source: Int J Environ Res Public Health. 2021 Jun 10;18(12):6302. doi: 10.3390/ijerph18126302 (PMC8296105; doi:10.3390/ijerph18126302)
Supplement: Supplementary file 1 [file ijerph-18-06302-s001.zip › Supplementary File S3 - FINAL.pdf]

File S3

S3a: Demographic characteristics of the 2013 census population, vision study sample and hearing study sample [12].

	2013 CENSUS POPULATION*	STUDY SAMPLE – VISION N=9188	STUDY SAMPLE – HEARING N=1393
	N (%)	N (%)	N (%)
**Age**			
Overall (aged 35+)	383,727 (100)	9188 (100)	1393 (100)
35 – 44	167595 (43.7)	4102 (44.7)	659 (47.3)
45 – 54	101183 (26.4)	2061 (22.4)	323 (23.2)
55 – 64	56894 (14.8)	1444 (15.7)	225 (16.2)
65 – 74	33755 (8.8)	1018 (11.1)	137 (9.8)
75 – 84	16521 (4.3)	441 (4.8)	38 (2.7)
85+	7779 (2.0)	122 (1.3)	11 (0.8)
**Sex**			
Male	192,969 (50.3)	2710 (29.5)	424 (30.4)
Female	190,758 (49.7)	6478 (70.5)	969 (69.6)
**Location**			
Urban	1,073,297 (57.8)	5039 (54.8)	906 (65.0)
Rural	783,884 (42.2)	4149 (45.2)	487 (35.0)

* Data from the 2013 census.

S3b: Demographic characteristics of the hearing survey responders and non-responders.

	HEARING RESPONDERS N=1393	HEARING NON- RESPONDERS N=944
	N (%)	N (%)
**Age**		
Overall (aged 35+)	1393 (100)	944 (100)
35 – 44	659 (47.3)	356 (37.7)
45 – 54	323 (23.2)	206 (21.8)
55 – 64	225 (16.2)	142 (15.0)
65 – 74	137 (9.8)	141 (14.9)
75 – 84	38 (2.7)	65 (6.9)
85+	11 (0.8)	34 (3.6)
**Sex**		
Male	424 (30.4)	245 (26.0)
Female	969 (69.6)	699 (74.0)
**Location**		
Urban	906 (65.0)	562 (59.5)
Rural	487 (35.0)	382 (40.5)
