# Supplementary material for: Estimating Need for Glasses and Hearing Aids in The Gambia: Results from a National Survey and Comparison of Clinical Impairment and Self-Report Assessment Approaches
Source: Int J Environ Res Public Health. 2021 Jun 10;18(12):6302. doi: 10.3390/ijerph18126302 (PMC8296105; doi:10.3390/ijerph18126302)
Supplement: Supplementary file 1 [file ijerph-18-06302-s001.zip › Supplementary File S4 - FINAL.pdf]

File S4

S4a: Clinical impairment assessment and self-reported unmet/undermet need for near and/or distance glasses (both mild and moderate vision impairment).

	**GLASSES UNMET/UNDERMET NEED***					
**Clinical impairment AD need**	***Clinical impairment assessment***					
	Distance glasses only	Near glasses only	Both distance and near glasses	*Total distance glasses*	*Total near vision glasses*	**TOTAL all glasses**
All glasses (mild/worse VI)	161	3634	371	*532*	*4005*	**4166**
All glasses (moderate/worse VI)	241	3821	184	*425*	*4005*	**4246**
**Washington group response**	***Self-reported***					
Some/worse difficulty (n=2530)			1681			**1681**
A lot/worse difficulty (n=179)			128			**128**

*Washington group data was data missing for 8 participants.

S4b: Clinical impairment assessment and self-reported unmet/undermet need for hearing aids (both mild and moderate hearing impairment).

**Clinical impairment AD need**	**HEARING AIDS UNMET/UNDERMET NEED**
	Clinical impairment assessment
Mild/worse HI	367
Moderate/worse HI	23
**Washington group response***	***Self-reported***
Some/worse difficulty (n=23)	17
A lot/worse difficulty(n=3)	3

* Comparison is limited to only hearing clinical impairment assessment survey participants (n=1393) for those reporting “some or worse” or “a lot or worse” difficulty hearing with or without hearing aids; Washington group survey responses for full survey participants (n=9180) are reported in Table 1, i.e. n=385 for “some or worse” difficulty hearing with or without hearing aids and n=55 for “a lot or worse” difficulty hearing with or without hearing aids
